# Influence of Smartphone-Based Digital Extension Service on Farmers’ Sustainable Agricultural Technology Adoption in China

**DOI:** 10.3390/ijerph19159639

**Published:** 2022-08-05

**Authors:** Baozhi Li, Ni Zhuo, Chen Ji, Qibiao Zhu

**Affiliations:** 1 Institute of Rural Development, Zhejiang Academy of Agricultural Sciences, Hangzhou 310021, China; 2 China Academy for Rural Development (CARD), Zhejiang University, Hangzhou 310058, China; 3 Department of Agricultural Economics and Management, School of Public Affairs, Zhejiang University, Hangzhou 310058, China

**Keywords:** smartphone-based digital extension service, sustainable agricultural technology adoption, soil testing formula fertilizer technology

## Abstract

The literature about how Information and Communication Technologies (ICTs) influence farmers’ adoption of sustainable agricultural technology is emerging, studies regarding the effects of particular smartphone-based digital extension services on farmers’ sustainable agricultural technology practices are limited. This study investigates the relationship between a digital extension service (“Zhe’ yang’ shi” WeChat application) and the adoption of soil testing and formula fertilization, a precision fertilization technology. A household choice model is constructed to explain the impact of the application. Based on a household-level data set from a survey of 400 farmers in Zhejiang in 2022, empirical results show that the use of the “Zhe’ yang’ shi” WeChat application significantly increases the adoption of soil testing and formula fertilization. We also discuss the heterogeneous effect by different production scales. The findings enrich the literature regarding ICTs’ influence on farmers’ behavior in adopting sustainable agricultural technology. It provides a valuable example for developing countries to promote sustainable agriculture through digital technology.

## 1. Introduction

Humanity has already transgressed or is rapidly approaching planetary boundaries [1], particularly greenhouse gas emissions, nitrogen and phosphorus pollution, and biodiversity reduction caused by agricultural production [2], consensus on accelerating the transformation of the food system, and fostering healthy soil are intensifying. Fertilizer is an important input to ensure the output of the food system, but the excessive input of chemical fertilizers causes soil acidification and weakens the productivity of crops [3]. The Paddy field ecosystem in southern China has been severely degraded due to excessive chemical inputs over the past few decades [4]. Technological progress is important to accelerate the transition towards a sustainable food system [5], such as promoting precision fertilization [6].

A growing body of literature highlights factors determining the adoption of precision fertilization and other sustainable agricultural technology. One strand of the literature focuses on the role of information because the assumption of complete information is usually difficult to hold up in practice; farmers often have limited access to information [7]. The lack and asymmetry of information will prevent farmers from adopting new technologies [8]. When farmers do not fully grasp the soil nutrient information, it is not only difficult to make the most efficient fertilizer allocation decisions [9], but also leads to soil acidification and other pollution [10,11]. In fact, there are many channels for farmers to obtain technical information, which have been studied and analyzed for technical training, agricultural extension services, farmer cooperatives, adoption of neighboring, social networking, advice cards, etc. [12,13,14,15,16,17,18,19]. Some literature measures the ability of information acquisition by summing up the number of acquisition channels [20] or calculating the probability of obtaining information through various channels [21]. On the one hand, the former ignores the heterogeneity of information acquisition channels; on the other hand, the latter has the problem of more random weight assignment caused by the prior value judgment of farmers [22].

In recent years, given that Information and Communication Technologies (ICTs) can overcome many limitations of traditional agricultural technology information promotion methods [23,24], the literature on ICTs affecting sustainable agricultural technology adoption is constantly emerging. They roughly include two lines. One is to emphasize that using ICTs reduces the cost of farmers’ information search and obtaining agricultural information in a timely manner, thus strengthening the farmers’ adoption of new technology [25,26]. Methods such as “face-to-face” interpersonal knowledge transfer are difficult to scale up sustainably, especially in highly decentralized smallholder farming systems [27] and against COVID-19 prevention and control. To this end, some literature analyzed the application of different ICTs to agricultural practices or technology adoption, such as audiovisual messages [28], toll-free hotlines [29], and short message services [30]. Campenhout et al. [27] analyzed the impact of adopting agricultural technology from three ICTs: video, intelligent voice reply, and SMS. Due to the Internet having more diversified and richer environmental information than traditional media [31], Li et al. [4] empirically discovered that smartphone use directly and positively affected farmers’ behavior toward adopting conservation agricultural technology.

Another thread emphasizes that digitalized ICTs can better provide personalized information and advice. For administrative convenience [32], the agricultural extension department’s traditional fertilization technology information services are often “blanket advice”, almost without considering the heterogenicity of the household and the environment. The effectiveness of traditional extension approaches has been limited because of information that is not sufficiently tailored to farmers’ needs [33,34], and the response to such recommendations has generally been poor [35]. Particularly for fertilization behavior, crop growth responses to fertilizers often vary significantly according to soil properties and nutrient levels [36,37]. In response to this challenge, mobile technology in decision support tools (DSTs) is considered an effective solution by greatly reducing the cost of delivering personalized extension services [38,39]. Aminou et al. [40] evaluate the effectiveness of a mobile application that provides personalized advice on rice nutrient management and found that farmers adopted an offsetting way to select the type and amount of fertilizer input according to actual needs. Rajkhowa & Qaim [7] show that using personalized digital extension services is positively and significantly associated with input intensity.

This study investigates the relationship between a digital extension service (“Zhe’yang’shi” WeChat application) and farmers’ adoption of sustainable agricultural technology (i.e., soil testing and formula fertilization, a precision fertilization technology). A household choice model is constructed to explain the impact of the application. Based on a household-level data set from a survey of 400 farmers in Zhejiang in 2022, empirical results show that the use of the “Zhe’ yang’ shi” WeChat application significantly increases the adoption of soil testing and formula fertilization both in terms of adoption amount and adoption land acreages.

This research has produced innovative work in the following aspects. First, the influence of personalized information on the application of precision fertilization technology is analyzed from both theoretical and empirical aspects. Previous studies have focused on the amount of information, but less on its attributes, especially on the personalization or heterogeneity of information on soil nutrients [7], which are key to determining technology adoption of precise fertilization [41]. Although few studies have focused on the personalized information supply of ICTs, it gives the soil nutrient information of a single crop, with less attention to the fertilization scheme information (for example, what kind and amount of fertilizer should be applied at different stages of farming). This is assuming that farmers can easily develop scientific fertilization schemes when they know the soil nutrient information. However, this assumption is difficult to satisfy in many practical situations. Therefore, it is necessary to explore the impact of richer personalized information on the adoption of precision fertilization technology for farmers, while little empirical evidence exists [9]. In addition, most studies consider the impact of information factors on the fertilizer amount, but in fact, the fertilization method or the amount of fertilizer under the scientific specification is more worthy of discussion. This is because only in the case of excessive fertilization, fertilizer reduction has a positive significance.

Second, the research has expanded from traditional ICTs to smartphone-based mobile Internet applications. One major reason for the difficulty of traditional ICTs in supplying personalized information is the technical limitations of information media. Mobile Internet applications are an essential feature that distinguishes smartphones from a feature phone, which is just a simple communication tool and other traditional ICTs. Those apps are profoundly changing the methods and results of farmers’ information acquisition, and their influence on the adoption of precision fertilization technology cannot be ignored. Mobile Internet applications based on digital technology advances are expected to improve the shortage of personalized information supply, while empirical evidence of actual impacts is scarce [7]. Previous studies focused more on the impact of smartphone use on farmers’ environmental behaviors in daily life and household welfare [42,43,44,45,46].

Third, this paper provides empirical evidence from the world’s largest developing country. It is essential to focus on the role of smartphones in environmental governance in developing economies [4]. However, previous studies on the impact of smartphone use on farmers’ precise fertilization behavior in developing countries have been poor. This study examines the influence of Zhe’ yang’ shi, a mobile Internet application emerging in China, on the adoption of soil testing and formula fertilization technology (Soil testing and formula fertilization technology: based on the soil test, according to the law of crop fertilizer demand and supply performance of soil fertilizer and reasonable use of nitrogen, phosphorus, potassium, and trace elements for crops. The central role of this technology is to solve the contradiction between the crop fertilizer demand and the soil fertilizer supply, to improve the fertilizer utilization rate and reduce the excessive application) for farmers to enrich the literature regarding ICTs influence on farmers’ sustainable agricultural behavior and provide implications for other developing countries to promote sustainable agriculture through digital technology.

The rest of this paper is structured as follows: Section 2 introduces the “Zhe’ yang’ shi” WeChat application and analytical framework. Section 3 explains the estimation methods and data used in this paper. Section 4 presents the empirical results and explanation. Section 5 provides the conclusions and discussion.

## 2. “Zhe’ yang’ shi” WeChat Application and Analytical Framework

### 2.1. Crop Production in Zhejiang and “Zhe’ yang’ shi” WeChat Application

Zhejiang province, located in southeast China, has very restrained natural resources (A common saying to describe the natural endowment of Zhejiang Province is “seven hills, one river, and two lands”, which means that Zhejiang Province consists of 70% hills, 10% water resources, and 20% arable lands). Crop production is the central department of agricultural production in Zhejiang Province. According to the Zhejiang Statistical Yearbook 2020, the output value of Zhejiang’s planting industry was CNY 159.396 billion (USD 23.559 billion), accounting for 45.58% of the total agricultural output value, and it has remained stable in the past 20 years. Regarding the planting industry’s output value, the proportions of grains, beans, and potatoes were 84.06%, 10.67%, and 5.27%, respectively. In addition, the top three crops with large planting areas in Zhejiang are grains, vegetables, and oilseeds.

Though natural resources in Zhejiang are restrained, the province is a leading region in China in terms of ICT adoption in agriculture. With the headquarter of digital and e-commerce giant in Zhejiang (i.e., Alibaba Inc. (Hangzhou, China)), and thousands of digital enterprises, Zhejiang province is an economically developed region famous for its ICT industry development. By the end of 2018, the average number of smartphones that each 100 rural households owned was 264 [47]. Digital and smart agriculture are widely applied in livestock, crop production, and infrastructure construction of villages. According to the “2021 National County-level Agricultural and Rural ICT Development Evaluation Report”, the overall ICT development level of Zhejiang ranked first in China in 2020 (66.7%), which overwhelmingly exceeded the national average level (37.9%).

**Figure 1:**
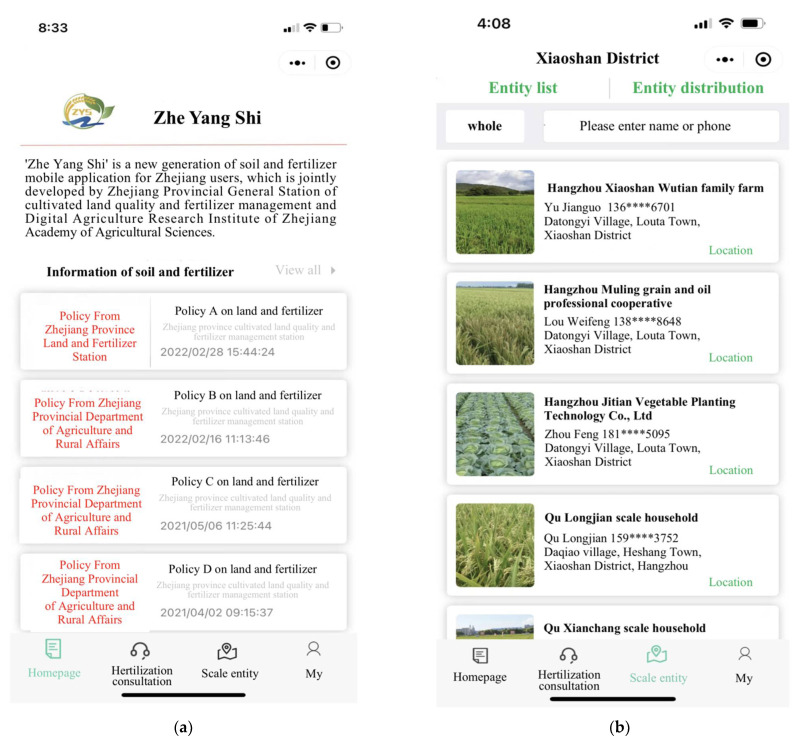

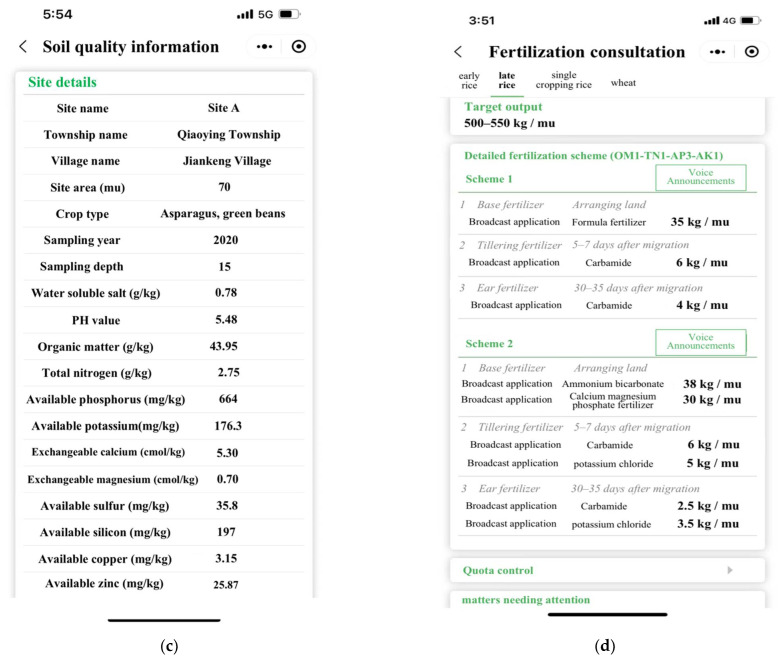
Screenshots of “Zhe’yang’shi” WeChat application. Notes: Subfigure (**a**) shows the screenshot of policy information, farmers can find the related policies on land and fertilizer. In subfigure (**b**), farmers can search their farm by entering names or phone numbers. Subfigure (**c**) shows the detailed soil quality information of every site. Subfigure (**d**) shows the fertilization consultation information, which provides farmers with the formula fertilization plans.

The government started to extend a smartphone-based WeChat application (named “Zhe’yang’shi”) to farmers in 2016, targeting the promotion of precision fertilization and farmers’ income. A new type of agricultural extension service, “Zhe’ yang’ shi” is designed and maintained by the agriculture department in Zhejiang Province and the Zhejiang Academy of Agricultural Sciences, providing information on soil nutrient and fertilization schemes at both village and product levels. Farmers can precisely adopt the advice for soil testing and formula fertilization simply by inserting their location and crop type in the application. The advice is generated from testing, calculating, and computing all soil in Zhejiang, based on 5G and big data analysis. Experimental counties started using “Zhe’ yang’ shi” in 2017, which was promoted at the provincial level in 2020. There are 12,600 households in 59 counties that are “Zhe’ yang’ shi” WeChat application users in Zhejiang Province. As the app has been embedded in the very popular local comprehensive application platforms such as “Zhejiang Office”, “Alipay”, and “WeChat”, the “Zhe’ yang’ shi” app does not even need to be downloaded separately. Thus, the free choice of multi-front-end is realized to facilitate different user needs. The following figures (Figure 1) are four screenshots of the “Zhe’ yang’ shi” WeChat application.

### 2.2. The Impact of “Zhe’ yang’ shi” on the Adoption of Soil Testing and Formula Fertilization

The soil testing formula fertilization behavior in this study refers to the adoption of soil testing and formula fertilization both in terms of adoption amount and adoption lands acreages. As a basic production unit, the decision-making process of peasant households on soil testing and formula fertilization behavior can be briefly understood as: under the constraints of production factors, the household trade-off between conventional fertilization (refers to the use of compound fertilizer with a fixed proportion of fertilizer) and soil testing and formula fertilization to pursue output maximization. The core contradiction is that soil testing and formula fertilization requires more specialized and personalized information (farmers need to receive and understand a large amount of information from each technology link when considering whether to adopt soil testing and formula fertilization technology [48]) than conventional fertilization and requires more labor due to multiple fertilization. Meanwhile, due to precision fertilization, soil testing and formula fertilization will have higher output efficiency and lower fertilizer costs and will receive government subsidies in line with the policy orientation of green agricultural development. Therefore, the behavioral decision model of soil testing and formula fertilization is constructed as follows:

$$Max\;Y(X_{1},X_{2})=P\cdot [Q_{1}(X_{1})+Q_{2}(X_{2})]+s\cdot X_{1}$$

$$s.t.\;K_{1}(X_{1})+K_{2}(X_{2})\leq K_{0}$$

$$L_{1}(X_{1})+L_{2}(X_{2})\leq L_{0}$$

$X_{1}$ represents the amount of soil testing and formula fertilization. $X_{2}$ represents the amount of conventional fertilization. $Q_{1}$ represents crop yields obtained by soil testing and formula fertilization. $Q_{2}$ represents crop yields obtained by conventional fertilization. P represents the unit price of crops. s represents subsidy coefficient of soil testing and formula fertilization. $K_{0}$ represents the budget for fertilizer input. $L_{0}^{}$ represents the amount of labor force used for fertilization. After distinguishing the use of funds for the purchase of fertilizer and the information on fertilization technology, and distinguishing the use of labor in the implementation of fertilization and obtaining the information on fertilization technology, the constraints can be represented as follows:

$$K_{1}+K_{2}=k_{1}+k_{2}+k_{i1}+k_{i2}=\alpha_{1}\cdot X_{1}+\alpha_{2}\cdot X_{2}+\delta_{1}\cdot X_{1}+\delta_{2}\cdot X_{2}$$

$$L_{1}+L_{2}=l_{1}+l_{2}+l_{i1}+l_{i2}=\beta_{1}\cdot X_{1}+\beta_{2}\cdot X_{2}+\gamma_{1}\cdot X_{1}+\gamma_{2}\cdot X_{2}$$

$\alpha_{1}$ represents the unit price of the fertilizer used for soil testing and formula fertilization. $\alpha_{2}$ represents the unit price of fertilizer used for conventional fertilization. $\delta_{1}$ represents the unit price of technical information on soil testing and formula fertilization. $\delta_{2}$ represents the unit price of conventional fertilization technical information. $\beta_{1}$ represents unit labor required for soil testing and formula fertilization. $\beta_{2}$ represents unit labor required for conventional fertilization. $\gamma_{1}$ represents unit labor to obtain the technical information on soil testing and formula fertilization. $\gamma_{2}$ represents unit labor to obtain technical information on conventional fertilization.

The Lagrange function is constructed as follows:

$$\begin{array}{l} L(X_{1},X_{2})=P\cdot [Q_{1}(X_{1})+Q_{2}(X_{2})]+s\cdot X_{1}\\ +\lambda_{1}(K_{0}-\alpha_{1}\cdot X_{1}-\alpha_{2}\cdot X_{2}-\delta_{1}\cdot X_{1}-\delta_{2}\cdot X_{2})+\lambda_{2}(L_{0}-\beta_{1}\cdot X_{1}-\beta_{2}\cdot X_{2}-\gamma_{1}\cdot X_{1}-\gamma_{2}\cdot X_{2}) \end{array}$$

The first-order necessary conditions to obtain the optimization using the Kuhn–Tucker theorem are:

$$L_{x1}=0=P\frac{\partial Q_{1}(X_{1})}{\partial X_{1}}+s-\lambda_{1}(\alpha_{1}+\delta_{1})-\lambda_{2}(\beta_{1}+\gamma_{1})$$

Let $\frac{\partial Q_{1}(X_{1})}{\partial X_{1}}=\frac{\lambda_{1}(\alpha_{1}+\delta_{1})+\lambda_{2}(\beta_{1}+\gamma_{1})-s}{P}=f(X_{1})$.

The equilibrium solution of soil testing and formula fertilization is obtained:

$$\tilde{X_{1}}=f^{-1}(\frac{\lambda_{1}(\alpha_{1}+\delta_{1})+\lambda_{2}(\beta_{1}+\gamma_{1})-s}{P})$$

Based on the universal assumption of diminishing marginal return, it can be considered $f(X_{1})$ as diminishing, and then its inverse function $f^{-1}(\cdot )$ is also decreasing. Therefore, in the case where parameters P, $\lambda_{1}$, $\lambda_{2}$ representing the price and the shadow price are greater than zero, the decrease in $\delta_{1}$ and $\gamma_{1}$ mean an increase in the $\tilde{X_{1}}$. So, the core of the problem becomes: What does using an app mean for the chance of $\delta_{1}$ and $\gamma_{1}$? Thanks to “Zhe’yang’shi”, soil nutrient information and fertilization schemes are accessible to users. Farmers only need to pay for the Internet fee and mobile phone power charges. In other words, government purchases reduce the unit price for farmers to buy technical information on soil testing and formula fertilization.

However, farmers have very little labor to get soil nutrient information and fertilization schemes from the app, as they just click on the location of their plots on the map. The required labor of monitoring the soil, developing fertilization schemes, maintaining systems, keeping information, and others, are undertaken by the government or a third party entrusted by the government. Farmers do not even have to download the app because it is already embedded in the popular social app (WeChat). In addition, the app also has a “voice broadcast” function for the fertilization scheme, thus further reducing the labor cost of aging farmers in accessing information. In conclusion, using the “Zhe’ yang’ shi” WeChat application can help farmers promote the adoption of soil testing and formula fertilization technology.

## 3. Research Design

### 3.1. Data and Descriptive Statistics

The dataset applied in this study is a household-level dataset from a survey of 400 farmers of Zhejiang province in 2022, which has detailed information on input, output volume, and value of crops, as well as the “Zhe’ yang’ shi” WeChat application adoption behavior of farmers (Please note that the whole data set is an annual longitudinal survey to 400 farmers of Zhejiang province from 2017 to 2021. However, we choose to use the 2021 cross sectional data (surveyed in 2022) in this study because the number of famers who started to use “Zhe’ yang’ shi” WeChat application in 2021 (56) and the accumulated number of farmers who adopted “Zhe’ yang’ shi” WeChat application in 2021 (148) are larger than any previous survey years). Four cities were randomly selected for this survey (Jiaxing, Shaoxing, Jinhua, and Taizhou). In each city, two counties were randomly selected. Five villages were randomly selected in each county, and ten crop farmers were randomly selected in each village. The Institute of Rural Development of the Zhejiang Academy of Agricultural Sciences conducted the surveys. Affected by the spread of the COVID-19 pandemic, this survey was conducted through online questionnaires by ten enumerators from the Zhejiang Academy of Agricultural Sciences; the enumerators were well-trained assistant researchers. The enumerators and the farmers could communicate in some WeChat groups when they encountered problems fulfilling the questionnaires. This study sets the following variables: Dependent variables are Acreage and Amount, which are the measurements of adoption of soil testing and formula fertilization in different dimensions. Independent variables include App, Gender, Age, Edu, Training, Pop, Land, Distance, Cooperatives, Brand, and Service. Table 1 shows the definition and descriptive statistics of the variables.

### 3.2. Econometric Model

The following linear regression model is constructed:

$$Y=\beta_{0}+\beta_{1}App+\beta_{2}Gender+\beta_{3}Age+\beta_{4}Edu+\beta_{5}Training+\beta_{6}Pop\;+\beta_{7}Land+\beta_{8}Distance+\beta_{9}Cooperatives+\beta_{10}Brand+\beta_{11}Service+\varepsilon$$

The definition and measurement of variables showed in Table 1. Where Y is the dependent variable using Acreage and Amount, respectively; $\beta_{0}$ is the intercept; (i = 1, 2, …, 11) are the coefficients of independent variables; ε is the disturbance term; independent variables include App, Gender, Age, Edu, Training, Pop, Land, Distance, Cooperatives, Brand, and Service. Ordinary least square (OLS) was used for estimating the coefficients.

## 4. Empirical Results

### 4.1. Effects of “Zhe’ yang’ shi” on Soil Testing and Formula Fertilization

Whether the control variables are added or not, the “Zhe’ yang’ shi” WeChat application can significantly promote soil testing and formula fertilization. In Table 2, columns (1) and (2) report the results of Ln (Acreage) as the dependent variable; columns (3) and (4) report the results of Ln (Amount) as the dependent variable. After adding the control variables, the coefficient of app in Column (2) is 0.7527, indicating that using the “Zhe’ yang’ shi” WeChat application can increase the acreage of land, which applied soil testing and formula fertilization technology by 75.27%. The coefficient of app in columns (4) is 0.7349, indicating that using the “Zhe’ yang’ shi” WeChat application can improve the amount of fertilizer, which applied soil testing and formula fertilization technology by 73.49%.

The gender, distance, and service significantly increase the adoption of soil testing and formula fertilization in the dimensions of acreage and amount. The head of the household being a male leads to a significant increase in the adoption of soil testing and formula fertilization. There are three instances where women become heads of the household in rural China [49]: one is a family where only a single mother and her children live together due to being divorced, widowed, or other reasons; the second is where the husband lost the ability to work because of illness or disability; the third is when the woman’s family has no male heir or husband in the family. These conditions mean that households with female heads have increased labor constraints and weaker risk resistance, preventing them from choosing more labor input and relatively unfamiliar techniques. Soil testing and formula fertilization require more time and types of fertilizer than conventional fertilization, so farmers are likely to go to agricultural stores more times. Staying away from agricultural materials stores will increase the corresponding labor burden. Farmers who have previously received the social services of soil testing and formula fertilization can better perceive the benefits of soil testing and formula fertilization, so they are more likely to adopt this technology.

### 4.2. Effects of “Zhe’ yang’ shi” on Different Farm Scales

To further investigate the effects of the “Zhe’ yang’ shi” WeChat application on different farm size farmers, this study divides the sample farmers into large-scale farmers group and small-scale farmers group according to their operating area. In Table 3, columns (1)–(2) report the results of Log(Acreage) as the dependent variable, and columns (3)–(4) report the results of Log(Amount) as the dependent variable. According to Xu et al. [50], in this paper, families operating an area above 3.33 hectares are defined as large-scale groups, and the others are small-scale groups.

Table 3 presents an interesting result: the group differences in the app coefficients were exactly opposite under the two dependent variables. When the dependent variable is Ln (Acreage), the app coefficient was not significant in the large group but significantly positive in the small group (1.2662). It means that the use of the “Zhe’ yang’ shi” app has increased the applied area by an average of 126.62%. When the dependent variable was Ln (Amount), the app coefficient was not significant in the small group but significantly positive in the large group (0.8478). It means that the use of the “Zhe’ yang’ shi” app has increased the applied weight in a large group by an average of 84.78%. The possible reasons are as follows.

First, when the dependent variable is Ln (Acreage), the coefficient of the app represents the percentage of applied area variation. If the app is to increase the area of soil testing and formula fertilization of large and small farmers by 1 unit, due to the difference in a land operated by the two, the percentage change of small farmers is bound to be higher. Moreover, compared with large farmers, small farmers are usually at a disadvantage when obtaining technical information on soil testing and formula fertilization, so the information gained by the app will be more obvious for small farmers. In addition, large farmers tend to apply soil testing and formula fertilization [51]. The applied area in a large group is probably already large before using the app, so the marginal contribution of the app is relatively small.

Second, when the dependent variable is Ln (Amount), the coefficient of the app represents the percentage of applied weight change. To some extent, applied weight versus applied area is a stricter measurement of the degree of soil testing and formula fertilization technology adoption because it considers not only the breadth of technology adoption but also the depth of technology adoption. Under the condition of the constant area, if the farmers’ understanding and mastery of the technology is relatively shallow, then the applied weight of soil testing and formula fertilization is relatively limited. The personalized and specialized information provided by the “Zhe’ yang’ shi” app helps farmers to understand and master the technology better, so it can help increase the applied weight of soil testing and formula fertilization. Due to the difference in operation scale, the above effect is more obvious in a large group.

## 5. Conclusions

The main work of this study was to investigate the relationship between mobile Internet applications and the adoption of precision fertilization technology through theoretical analysis and empirical testing. After a brief introduction of the “Zhe’ yang’ shi” WeChat application, a new generation of digital mobile terminal platform emerging in China, a household choice model was constructed to explain the app’s impact on the adoption of soil testing and formula fertilization, which is a precision fertilization technology. Based on a household-level dataset from a survey of 400 farmers of the Zhejiang province, empirical results show that the use of the “Zhe’ yang’ shi” WeChat application increases the adoption of soil testing and formula fertilization. Further, the effect varies in different scales of operation: there is an increase in the applied area in the small group, but an increase in applied weight in a large group. The findings of this study enrich the literature regarding the impact of ICTs on farmers’ sustainable agricultural technology adoption by uncovering the advantages of mobile Internet apps in strengthening the information support of precision fertilization technology.

The rationality and legitimacy of the “Zhe’ yang’ shi” WeChat application as a public product is the positive externality of soil testing and formula fertilization technology. The technology is conducive to cultivating healthy soil and reducing environmental pollution by promoting precise fertilization, so it belongs to an agricultural green technology or sustainable development technology. To promote the application of the technology, the government needs to minimize the difference between farmers’ private and social costs or between farmers’ private and social benefits. Although there have been corresponding government actions in the past, it has mainly been cash subsidies. As mentioned above, adopting soil testing and formula fertilization technology is dependent on information, and purchasing information with specialty and heterogeneity through the market requires high fees. Therefore, simple cash subsidies are difficult to work, or the amount of subsidies needed is huge, which increases the financial burden. Therefore, the emergence of the “Zhe’ yang’ shi” WeChat application reflects a new idea of expanding the supply of public goods needed to promote agricultural green technology, from cash subsidies to information support.

Second, it can reduce the unit labor to obtain information. In addition to purchasing, farmers may also obtain technical information on soil testing and formula fertilization through labor. Two main ways are to directly search for and obtain their own soil nutrient information and the corresponding fertilization scheme. However, this is not an easy thing in ordinary channels. TV, computer, radio, books, training, feature phone, and other ordinary channels spread ideas, basic principles, successful cases, market information, etc., aiming to awaken or strengthen farmers’ awareness of environmental services [52,53], while the practical technical information with both specificity and heterogeneity is rare. Farmers need to search through a vast ocean of information for their own “pearls”, and it is usually unclear where to get them. Higher difficulty in obtaining information means higher labor costs.

Another indirect acquisition of the technical information is to obtain some empirical methodology of soil testing and formula fertilization first, and subsequently applying it to their land. A common way is to consult experienced farmers, learn how they perceive soil nutrient information, and develop a fertilization scheme based on experience. Even if they can be consulted through ICTs such as phone calls and SMS [54], it is difficult to obtain effective information. Because this information is based on subjective experience about a specific time and place belongs to the tacit knowledge or decentralized knowledge. In the eyes of the Hayek sect, such a message is barely transmitted. The solution to making good use of this tacit knowledge given by the Hayek school is to take the market price system as an information exchange mechanism so that individuals in this system only need to know the scattered local information to act independently and harmoniously with each other [55]. However, this method is ineffective in the information of soil testing and formula fertilization technology because the high transaction cost hinders the birth and operation of the information market. Moreover, the price signal of the fertilizer market can only guide farmers’ decisions on the amount of used fertilizer or can only improve their willingness to conduct precise fertilization. Still, it is difficult to guide the behavior of soil testing and formula fertilization directly.

Based on the advantages of digital technology, the “Zhe’ yang’ shi” WeChat application can take tacit knowledge into explicit knowledge and reduce the unit labor of information acquisition by reducing the difficulty of information transmission. Different from Hayek’s idea of taking a market price system as a communication mechanism, the “Zhe’ yang’ shi” WeChat application is similar to information Keynesian [56], emphasizing the “visible hand” of the government, which solves the problem of missing information market of soil testing and formula fertilization. Of course, the “Zhe’ yang’ shi” WeChat application is also different from the traditional information public goods. It can solve the contradiction between the general supply and personalized demand of information public goods, such as by fully displaying the real preferences of users. To sum up, the practice and experience of the “Zhe’ yang’ shi” WeChat application provide a valuable reference for other developing countries to accelerate the promotion of precision fertilization technology through new ICTs, and then improve the efficiency of fertilizer and reduce fertilizer pollution.

## Figures and Tables

**Table 1:** Definition of the variables and descriptive statistics.

Variables	Definition	Mean	Std. Dev.	Min	Max
*Dependent variables*					
Acreage	Acreage of land that applied soil testing and formula fertilization technology (unit: mu)	415.911	1211.894	0	10,150
Amount	Amount of fertilizer that applied soil testing and formula fertilization technology (unit: ton)	16.893	29.973	0	180
*Independent variables*					
App	Household usage of Zhe’yang’shi 1 = used in 2021, 0 = otherwise	0.37	0.4834	0	1
Gender	Gender of household head: 1 = male, 0 = female	0.71	0.454	0	1
Age	Age of household head (unit: years)	42.75	11.628	20	72
Edu	The schooling years of household head (unit: years)	11.35	4.051	1	24
Training	Whether received training about agricultural green technology 1 = yes, 0 = no	0.66	0.474	0	1
Pop	Number of people residing in a household	4.85	1.886	1	15
Land	Total area of agricultural land operated by household (unit: mu)	547.73	1407.359	1	12,000
Distance	Distance to the nearest fertilizer store (unit: km)	10.15	12.834	0	70
Cooperatives	Whether household has an agricultural cooperative member 1 = yes, 0 = no	0.52	0.500	0	1
Brand	Whether to have an agricultural public brand 1 = yes, 0 = no	0.56	0.497	0	1
Service	Whether accepted the socialized service of soil testing and formula fertilization 1 = yes, 0 = no	0.53	0.499	0	1

Note: Mu is a traditional land area unit in China. 1 mu = 1/15 hectare.

**Table 2:** Effects of “Zhe’ yang’ shi” on soil testing and formula fertilization.

	Ln (Acreage)		Ln (Amount)	
	(1)	(2)	(3)	(4)
App	1.2850 *** (0.2627)	0.7527 *** (0.2610)	0.8679 *** (0.1518)	0.7349 *** (0.1539)
Gender		1.2353 *** (0.3166)		0.6497 *** (0.1868)
Age		−0.0046 (0.0137)		−0.0085 (0.0081)
Edu		−0.0236 (0.0363)		0.01246 (0.0214)
Training		0.4253 (0.3253)		−0.1916 (0.1918)
Pop		0.0109 (0.0626)		−0.0354 (0.0369)
Land		0.0001 (0.0000)		0.0002 *** (0.0000)
Distance		0.0317 *** (0.0099)		0.0226 *** (0.0058)
Cooperatives		0.5367 * (0.2871)		−0.1060 (0.1694)
Brand		−0.0955 (0.2622)		0.2922 * (0.1546)
Service		0.6201 * (0.2619)		0.4075 *** (0.1545)
_cons	2.8267 *** (0.1598)	1.3334 (0.9100)	1.4657 *** (0.0923)	0.9348 * (0.5368)
Obs	400	400	400	400
R^2^	0.0567	0.2253	0.0759	0.2089

Note: Standard errors in parentheses. * *p* < 0.1, *** *p* < 0.01.

**Table 3:** Effects of “Zhe’ yang’ shi” on different scales.

	Log (Acreage)		Log (Amount)	
	(1) Large-Scale	(2) Small-Scale	(3) Large-Scale	(4) Small-Scale
App	0.4379	1.2662 ***	0.8478 ***	0.1109
	(0.39)	(0.29)	(0.21)	(0.25)
Gender	1.0620	1.6480 ***	0.7986 *	0.3934 **
	(0.79)	(0.23)	(0.44)	(0.19)
Age	−0.0091	0.0170	−0.0007	−0.0232 *
	(0.02)	(0.02)	(0.01)	(0.01)
Edu	−0.0547	0.0355	0.0172	−0.0170
	(0.07)	(0.03)	(0.04)	(0.03)
Training	0.6006	0.7402 **	−0.0088	−0.0774
	(0.59)	(0.28)	(0.33)	(0.24)
Pop	−0.2116 *	0.0601	−0.1402 **	0.1000 **
	(0.12)	(0.05)	(0.06)	(0.04)
Land	0.0002 *	−0.0019	0.0002 ***	0.0135
	(0.00)	(0.01)	(0.00)	(0.01)
Distance	0.0404 ***	−0.0158	0.0238 ***	−0.0019
	(0.01)	(0.02)	(0.01)	(0.01)
Cooperatives	0.4070	0.8882 ***	−0.4081	0.4911 **
	(0.48)	(0.26)	(0.27)	(0.22)
Brand	−0.0611	0.2628	0.1746	0.7300 ***
	(0.40)	(0.27)	(0.22)	(0.23)
Service	0.8907 **	−0.1719	0.3664	0.0975
	(0.42)	(0.27)	(0.23)	(0.23)
_cons	2.7079 *	−0.1582	0.8542	1.2600 *
	(1.62)	(0.87)	(0.89)	(0.74)
Obs	248	152	248	152
R^2^	0.1470	0.6240	0.2388	0.2997

Note: Standard errors in parentheses. * *p* < 0.1, ** *p* < 0.05, *** *p* < 0.01.

## Data Availability

The data presented in this study are available on request from the corresponding author. The data are not publicly available.
